# Clinical and Functional Outcomes of Anterior Cruciate Ligament Reconstruction at a Minimum of 2 Years Using Adjustable Suspensory Fixation in Both the Femur and Tibia: Letter to the Editor

**DOI:** 10.1177/23259671241294052

**Published:** 2025-01-31

**Authors:** Ranjeet Choudhary, Ankit Kumar Garg, Nagaraju Venishetty

**Dear Editor:**

We read with interest a study by Colombet et al^2^ that was published in your journal. The authors prospectively followed a cohort of 97 patients who underwent anterior cruciate ligament reconstruction with quadrupled semitendinosus autograft using adjustable cortical suspensory fixation devices. They evaluated the side-to-side difference in anterior laxity, as well as clinical scores (International Knee Documentation Committee and Lysholm score) preoperatively and at 6, 12, and 24 months postoperatively. A regression analysis was performed to determine the association between the outcomes and 9 preoperative independent variables. It is an excellent study, highlighting the efficacy of adjustable cortical fixation devices in graft fixation, with good outcomes at long-term follow-up.

However, we found some discrepancies in the statistical tests used in the study. The authors evaluated improvements in knee laxity and clinical scores at consecutive follow-ups using the Mann-Whitney test and overall trends using the Kruskal-Wallis test. The Kruskal-Wallis and the Mann-Whitney tests are used as tests for significance in non-Gaussian distribution with unpaired sample groups.^1,3,4^ Since the authors compared the paired samples (knee scores at consecutive follow-ups), it would have been ideal to use the Wilcoxon rank-sum test (2 paired groups) and the Friedman test (>2 groups with paired samples). We believe that it may be some typographical error.


Ranjeet Choudhary, MBBS, MS (Ortho)
Ankit Kumar Garg, MBBS, DNB (Ortho), Dip. SICOT
Nagaraju Venishetty, MBBS, DNB (Ortho) *Raipur, India*

